# OnabotulinumtoxinA Is an Effective Treatment for Chronic Migraine in Patients With Comorbid Fibromyalgia

**DOI:** 10.3389/fneur.2020.575130

**Published:** 2020-10-15

**Authors:** María Sastre Real, Javier Díaz de Terán

**Affiliations:** ^1^Department of Neurology, La Paz University Hospital, Madrid, Spain; ^2^La Paz Institute for Health Research (IdiPAZ), Madrid, Spain; ^3^CranioSPain Research Group, La Salle Higher Center for University Studies, Physiotherapy Department, Autonomous University of Madrid, Madrid, Spain

**Keywords:** chronic migraine, fibromyalgia, onabotulinumtoxinA, central sensitization, comorbidities, quality of life

## Abstract

**Introduction:** Fibromyalgia (FM) is a frequent comorbidity in patients with chronic migraine (CM). PREEMPT trials, which demonstrated the efficacy of OnabotulinumtoxinA (OnabotA) on CM, excluded patients with FM. Our aim was to evaluate the effectiveness of OnabotA in a series of patients with CM and FM.

**Methods:** We analyzed patients with a previous diagnosis of CM and FM who had received sessions of OnabotA quarterly between January 2014 and January 2020 in a specialized Headache Clinic. Primary endpoint was the reduction in moderate to severe headache days at 3, 6, 9, and 12 months.

**Results:** Data were collected from 31 patients with CM and FM that received OnabotA (100% females). Mean age at first procedure was 50.2 ± 11.3 years. Depression (93.5%), other central sensitization syndromes (irritable bowel syndrome, interstitial cystitis, multiple chemical sensitivity, endometriosis, and chronic fatigue syndrome) (48.4%), and medication overuse headache (90.3%) were frequent comorbidities. 48.4% of patients had failed ≥3 preventives previously. The percentage of patients who achieved ≥30 and ≥50% moderate-severe headache reduction on the third month was 65.4 and 48.2%, respectively. Twenty-three patients completed four cycles of treatment, with 13.4 fewer headache days per month than at baseline (*p* < 0.001). By 1 year, 69.5% had ≥50% reduction of headache frequency and 39.1% had a ≥75% reduction. In 4 cases (21%), OnabotA was interrupted due to a lack of response. Only mild adverse effects were recorded.

**Conclusion:** OnabotA is an effective treatment for CM in patients with FM.

## Introduction

Chronic migraine (CM) and fibromyalgia (FM) are prevalent and disabling pain disorders that frequently coexist (1). CM is defined as headache occurring on 15 or more days/month for more than 3 months, which, on at least 8 days/month, has the features of migraine headache (2). CM affects 2% of the general population and results in substantially greater disability than episodic migraine. Patients with CM are also more likely to have comorbid disorders such as depression, anxiety, and other chronic pain (3).

FM is a chronic pain syndrome characterized by diffuse pain in addition to disturbed sleep, cognitive impairment, and/or pronounced fatigue (4, 5). Many studies have reported high rates of FM in patients with migraine, that range between 10 and 31% in patients with episodic migraine (6–12), and from 37 to 80% in patients with CM (9, 10, 13, 14). Migraineurs with comorbid FM report more depressive symptoms, higher headache intensity, and more disability (15).

Central sensitization has been purposed to be the common mechanism in both FM and migraine pathogenesis (6, 16–21). Cutaneous allodynia, low pain thresholds, and dysfunction of brainstem and cortical areas involved in the modulation and processing of pain have been found in patients with CM (22–24). Taken together, this suggests that central pain processing dysfunction may be linked to both migraine chronicity and spreading of pain that results in development of FM. Other central sensitization syndromes are irritable bowel syndrome, interstitial cystitis, multiple chemical sensitivity, endometriosis, and chronic fatigue syndrome.

OnabotulinumtoxinA (OnabotA) is a treatment specifically approved for the prophylaxis of CM. Its efficacy has been demonstrated in two large Phase 3 trials (PREEMPT 1 and 2), together enrolling 1,354 individuals (25, 26). Subsequent analyses showed consistent results and a cumulative benefit over time (27, 28). Treatment of CM with OnabotA was associated with a sustained reduction in headache frequency, reduction in acute medications, and significant improvement in quality-of-life and disability measures. Moreover, symptoms of depression, anxiety, poor sleep and fatigue were all improved (27–34). Several studies demonstrated that 60–80% of patients reported a long-term reduction in headache days and/or migraine days of at least 50% from baseline (35–39).

Patients with FM were excluded from the PREEMPT trials (25, 26). Even though FM is a frequent comorbidity in patients with CM and contributes to disability, studies that analyze the response to preventive treatments in this subgroup of patients have been lacking (40). To the best of our knowledge, no studies designed to evaluate the response to OnabotA for CM in patients with FM have been published. The aim of the present study is to assess the effectiveness of OnabotA in this group of patients.

## Materials and Methods

This was a retrospective, observational study carried out in the city of Madrid, Spain, at a specialized Headache Clinic. We conducted a review of patient data accumulated in the normal conduct of standard medical practice. No prospective treatment assignments were made, and all assessments were routine standard of care. The study was reviewed and approved by the local Ethics Committee for Clinical Research of a public reference hospital (PI-3882). All patient data were treated with confidentiality, in accordance with the Declaration of Helsinki (41).

Subjects with FM were selected for recruitment from a database of patients that attended our Headache Clinic between June 2019 and January 2020. We reviewed the medical charts of those patients from January 2014 to January 2020. Subjects were included in this study if they met the following requirements: (1) had a diagnosis of CM according to the International Classification of Headache Disorders 3 (ICHD-3) criteria; (2) had received a diagnosis of FM from a rheumatologist following the 2010 criteria of the American College of Rheumatology; and (3) had received injections of OnabotA for the treatment of CM between January 2014 and January 2020.

Patients were excluded if they had a previous diagnosis of primary or secondary headache that could interfere with the effectivity measures, neuromuscular disease, or any contraindications for treatment with OnabotA.

Patient demographic data, headache history, and clinical features were obtained through a standardized oral questionnaire at baseline. Data regarding acute and prophylactic medications for headache or for other conditions were also documented.

OnabotA was initiated in patients who had either not responded to at least two oral preventive treatments or had contraindications or risk factors precluding the use of oral prophylactic medications. Subjects received intramuscular OnabotA injections following the PREEMPT paradigm (155–195 units in 31–39 sites) every 3 months (±2 weeks). The response to this treatment was evaluated quarterly. Prior preventative treatments were continued at the discretion of the treating neurologist. Success was defined as a ≥ 30% reduction in headache days per month or a clinically significant improvement of headache disability reported by the patient. OnabotA therapy was discontinued if treatment success was not achieved over the first three treatment cycles.

As per usual clinical practice, patients maintained a paper or electronic diary during the OnabotA treatment period to register headache days and acute medication intake. The first year of treatment (four cycles) was considered to analyze the effectiveness and tolerability of OnabotA (42). The primary endpoint was reduction in moderate to severe headache frequency at 3, 6, 9, and 12 months in accord with the Guidelines of the International Headache Society for controlled trials of preventive treatment of CM in adults. A moderate/severe headache day was defined as a day with moderate or severe pain lasting at least 4 h or a day with a headache that was successfully treated by an acute headache medication (43). We considered moderate to severe headache as the primary endpoint because it is difficult for the patients to register all headache features for each attack and these definitions allow the use of a relatively simple headache diary.

We used ANOVA (significance level ≤ 0.05) to test for statistical significance of reported headache days per month between baseline and at 3, 6, 9, and 12 months. Statistical analyses were performed with IBM SPSS Statistics 25.0 (IBM, Armonk, NY).

## Results

We included 31 patients diagnosed with CM and concomitant FM that met eligibility criteria. Demographic and baseline patient characteristics are provided in Table 1. At least three prior preventive treatments had failed in about half of the patients (48.4%).

**Table 1:** Demographic and baseline clinical characteristics.

Variables	*N* = 31
Age (years) (mean, SD)	50.2, 11.3
Gender (*n*, %)	
Female	31 (100)
Male	0 (0)
Comorbidities (*n*, %)	
Depression	29 (93.5)
Medication overuse headache	28 (90.3)
Other central sensitization syndromes^*^	15 (48.4)
Other chronic pain syndrome	27 (87.1)
Autoimmune disease	12 (38.7)
Migraine duration (years) (mean, SD)	24.5, 15.7
Moderate/severe headache days per month (mean, SD)	24.9, 5.3
Prior failed preventive treatments (*n*, %)	
≥2	27 (87.1)
≥3	15 (48.4)
≥4	10 (32.3)
Concomitant preventive treatment at first injection (*n*, %)	23 (74.2)

SD = standard deviation.

* *Irritable bowel syndrome, interstitial cystitis, multiple chemical sensitivity, endometriosis, and chronic fatigue syndrome*.

Among the 31 patients included, 23 (74.2%) were receiving a concurrent oral preventive migraine therapy when OnabotA was initiated. Sixteen patients (51.6%) were on antidepressants. Twenty-eight patients (90.3%) fulfilled medication overuse headache (MOH) criteria according to ICHD-3, including 21 (67.7%) patients overusing opioids. Interestingly, only nine patients (29%) overused analgesics solely to alleviate headache and only five patients (16.1%) were triptan abusers. Most analgesic abusers (67.9%) and opioids abusers (80.9%) were consuming acute medications mainly to mitigate extracranial pain due to FM or pain conditions other than migraine.

**Figure 1:**
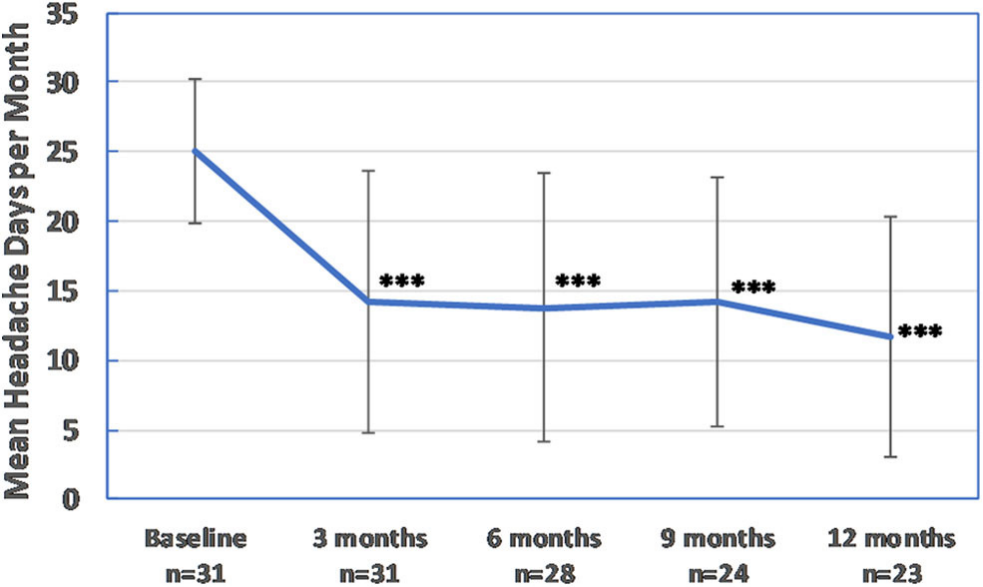
Effectiveness of OnabotA: moderate to severe headache days per month. Error bars are ±1 standard deviation. ***p < 0.001 vs. baseline.

**Figure 2:**
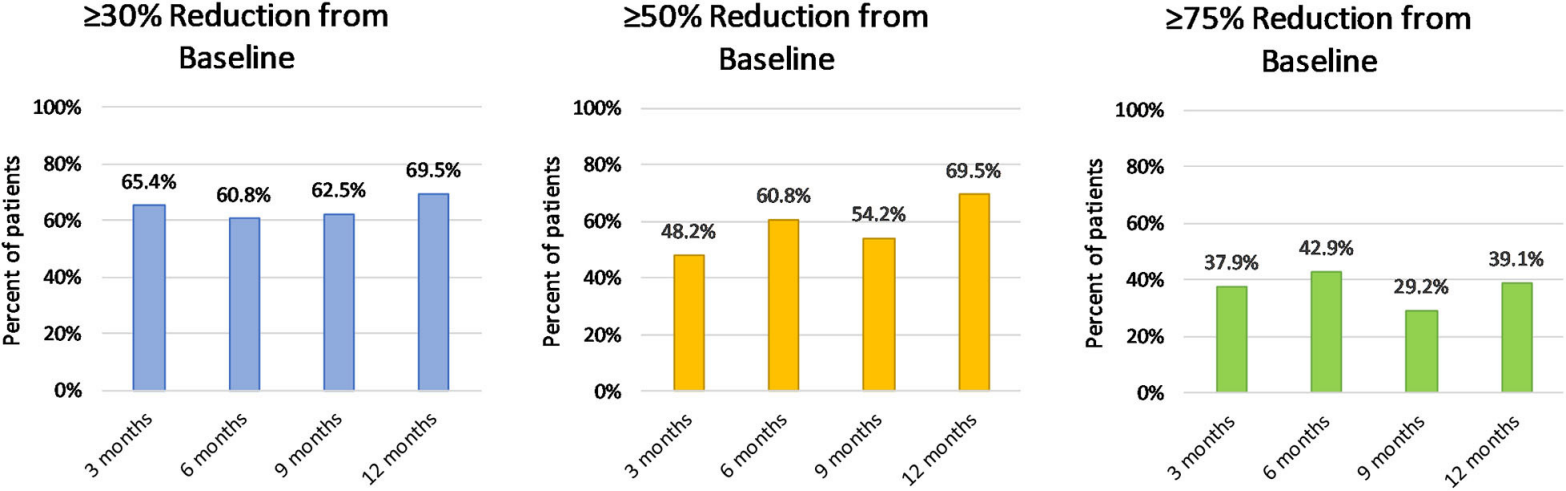
Effectiveness of OnabotA: proportion of patients with ≥30, ≥50, and ≥75% reduction of moderate to severe headache days per month.

A ≥30% reduction in headache days per month was achieved by 65.4% of patients at 3 months; 48.2% of patients achieved a ≥50% reduction, and 37.9% of patients achieved a ≥75% reduction. After one cycle of treatment, mean moderate to severe headache days were significantly reduced from 24.9 ± 5.2 to 14.2 ± 9.4 (*p* < 0.001). A total of 23 patients received at least four treatment cycles. After four cycles, 69.5% of patients experienced a reduction of at least 50% in moderate to severe headache days per month, and 39.1% reported a reduction of ≥75%. Moderate to severe headache days per month were reduced from 25.1 ± 5.2 to 11.7 ± 8.6 (*p* < 0.001). All patients with ≥50% response after one cycle continued being responders after 1 year of treatment. Treatment outcomes during 1 year of treatment are summarized in Figures 1, 2. OnabotA was discontinued in four patients (12.9%) due to a lack of response. Four patients began treatment after January 2019 and were continuing treatment with <1 year of follow-up at the time of data analysis.

None of the patients discontinued their oral migraine prophylactic treatments. Of the 28 patients that fulfilled criteria for MOH at baseline, the condition was solved for 18 (64.3%), reducing the prevalence from 90.3 to 32.3%. Nevertheless, 24 patients continued to consume excessive medication (≥10 days of triptans, ≥15 days of NSAIDs, or ≥10 days of opioids per month) for other pain conditions.

There were few reported adverse events for OnabotA and none were considered serious. Reported adverse events were ptosis in one patient (3.2%) and mild vagal reaction in one patient (3.2%).

## Discussion

CM is a prevalent and disabling neurologic disorder that is often associated with comorbid conditions that can influence both its prognosis and its impact on quality of life (1, 3, 23).

Several studies have reported a high prevalence (up to 80%) of FM in patients with CM (9, 10, 13, 14), and there is a large body of evidence that central sensitization comprises the common pathophysiologic basis (1). FM and migraine when present together contribute to increased disability. Furthermore, the presence of FM often makes the management of CM less straightforward owing to the presence of concomitant treatments, widespread pain with the increased risk of analgesic abuse, and greater prevalence of psychiatric comorbidity.

Patients with both CM and FM require a tailored and multidisciplinary treatment approach. PREEMPT clinical trials and subsequent real-life studies have demonstrated the efficacy, safety, and tolerability of OnabotA as a prophylactic treatment for CM in adults (25, 27, 30, 35–37). However, patients with FM were excluded from the PREEMPT trials. Given the high prevalence of FM in CM patients, the lack of studies that evaluate responsiveness to CM treatments, and in particular to OnabotA, in this group of patients is surprising (40). Two Spanish studies that evaluated the effectiveness of OnabotA for CM included FM among other variables in their analysis and reported that FM did not significantly influence the rate of response to OnabotA (36, 37). However, the included FM patients comprised a relatively small subset of the study populations, and detailed assessment of response was not reported for these patients with FM. To the best of our knowledge, this is the first study that specifically assesses the effectiveness of OnabotA for CM in a cohort of patients with FM.

Our results show that OnabotA is effective in patients with CM and FM. After 1 year of treatment, 69.5% of patients reduced their moderate to severe headache frequency by at least 50% and a mean headache days reduction of 13.4 ± 10.0 (*p* < 0.001) was observed. This high response rate was in spite of the presence of several negative predictive factors such as long duration of CM, multiple prior preventive treatment failures, and several comorbidities. Importantly, 74.2% of patients were treated for at least 1 year, minimizing the potential confounding influence of natural fluctuations in the disease course itself (42). The 1 year span of treatment highlights the long-term safety and tolerability of OnabotA, even in the presence of comorbidities. Our results are consistent with those of other real-world published studies on CM in terms of main efficacy measures (33, 35–37). However, other studies have reported high rates of concomitant prophylactic treatment discontinuation and reduction of acute medication intake with OnabotA (33), which was not observed in our study. It is worth emphasizing that most concomitant prophylactic medications used in our sample had a dual indication: both for migraine and depression or for migraine and concomitant extracephalic chronic pain conditions. Therefore, it is not surprising that patients continued to need those treatments, regardless of their migraine improvement. On the other hand, the vast majority of patients took acute medications (opioids in particular) to relieve other pain conditions (FM and other chronic pain syndromes). OnabotA significantly reduced headache frequency even though most of these patients continued to use excessive medication. Indeed, MOH reduced from 90.3 to 32.3%. This finding, consistent with previous studies, confirms that OnabotA is effective in patients with MOH who do not receive withdrawal interventions (44–46). Thus, in patients with FM, even when reduction of acute medication use is not the short-term objective, headache frequency can be reduced without acute medication withdrawal. This finding reinforces the idea that the presence of FM, even with the abuser condition, should not preclude use of OnabotA.

The limitations of the present study include the retrospective, single-center design with a relatively small sample size, and the lack of a control group. Nevertheless, the efficacy results are consistent with those of large prospective observational studies. While the relatively “open” inclusion criteria could introduce variability, it was, in our view, a key strength. While randomized controlled trials have inarguable intrinsic validity, “real-world” data such as the cohort described herein have extrinsic validity.

The treatment of migraine should not be so narrow that comorbid conditions impinging on quality of life are ignored. FM evaluation and treatment should be considered in the daily practice of patients with migraine (47). This study showed OnabotA to be a safe and effective treatment for CM in patients with FM. Additional studies confirming these results are warranted.

## Data Availability Statement

The raw data supporting the conclusions of this article will be made available by the authors, without undue reservation.

## Ethics Statement

The studies involving human participants were reviewed and approved by Comité de Ética de la Investigación con medicamentos del Hospital Universitario La Paz. Written informed consent for participation was not required for this study in accordance with the national legislation and the institutional requirements.

## Author Contributions

MS created the original manuscript draft and performed the data analysis. JD revised the original draft manuscript. MS and JD contributed equally to the conceptualization and design of the study. All authors contributed to the article and approved the submitted version.

## Conflict of Interest

The authors declare that the research was conducted in the absence of any commercial or financial relationships that could be construed as a potential conflict of interest.
